# Family-based analysis of a myocardial infarction endophenotype: comparison of sampling designs

**DOI:** 10.1186/1753-6561-3-s7-s120

**Published:** 2009-12-15

**Authors:** Chengrui Huang, Ke Li, Rose Saint Fleur, Su-Wei Chang, Seung Hoan Choi, Tong Shen, So Youn Shin, Stephen J Finch, Nancy R Mendell

**Affiliations:** 1 Department of Applied Mathematics and Statistics, Math Tower 1-111, State University of New York at Stony Brook, New York 11794-3600, USA

## Abstract

The power of linkage analysis of a quantitative disease endophenotype was compared for the following family selection designs: 1) Random samples: randomly chosen nuclear families, 2) "coronary artery calcification (CAC)" samples: selection of each nuclear family through a proband with abnormally high levels of the simulated quantitative endophenotype, CAC, and (3) "MI" samples: selection of each nuclear family through a disease affected proband, in this case a proband who had been simulated to have a myocardial infarction (MI) event.

We assessed the power to detect linkage to five loci (two pairs of epistatic loci and one locus with an over-dominant allele) that were modeled as determinants of the simulated CAC levels. We did this using a Haseman-Elston regression-based linkage analysis of the adjusted CAC levels that considered each locus separately and then used a multiple regression extension of the Haseman-Elston method in which we considered the allele sharing at two true epistatic loci simultaneously and their interaction as possible factors related to the squared sibpair differences in adjusted CAC.

Based on comparison of the mean square root of the LOD scores, there was no one sampling design that resulted in consistently greater power for these five loci. That is, we observed significant locus-by-sampling-design interaction (*p *< 0.0001). We noted however, that the largest average  score was observed for the epistasis between *τ*_3 _and *τ*_4 _(mean  > 1.8, SE = 0.06) in the MI-selected samples and the CAC-selected samples.

## Background

It is well established that the selection of probands, or sibpairs, with extreme phenotypes in small nuclear families increases power over random sampling for detection of linkage with quantitative trait loci [1,2]. Sibpairs selected so that their phenotypes are discordant (i.e., one with a high value and one with a low value) generally provide the largest gains in statistical power, but such families require much larger samples for phenotypic pre-screening.

We are usually interested in a quantitative trait because of its association with some disease. Sometimes the quantitative trait of interest is a clinical measure that more accurately captures the extent of the disease than the disease diagnosis itself, but often the quantitative trait is a risk factor for some complex disease, and hence a disease endophenotype. Thus, one would expect that a linkage analysis of a quantitative trait based on sibpairs in which there is at least one disease-affected individual would be more powerful than an analysis based on randomly selected sibpairs. Sung et al. [3] refers to such a sampling design as asymmetrically ascertained sibpairs (AASP) in a recent paper in which they developed numerical methods for evaluating the power of a linkage analysis of qualitative endophenotype using AASP.

In this paper we compared three methods for sampling nuclear families for linkage analysis of a quantitative endophenotype. The three methods we considered were random selection of nuclear families, selection of nuclear families through probands simulated as having the complex disease, and selection of nuclear families through probands who had high values of the disease-associated quantitative endophenotype.

We analyzed the Problem 3 data provided by the Genetic Analysis Workshop 16. The genotype and pedigree structure of the individuals in the data set were provided by the Framingham Heart Study. However, all phenotypic information was simulated based on the models described in Kraja et al. [4]. The quantitative endophenotype we consider was called coronary artery calcification (CAC), which is a major risk factor for the simulated complex disease, myocardial infarction (MI). For our analysis we considered the five single-nucleotide polymorphisms (SNPs) (denoted *τ*_1_, *τ*_2_, *τ*_3_, *τ*_4_, and *τ*_5_), which had genotypes modeled to have direct effects on the simulated CAC values and five SNPs (rs10044327 denoted M1, rs3776649 - M2, rs32609 - M3, rs12152770 - M4, rs6887019 - M5) on chromosome 5. Two pairs of these genes, *τ*_1 _with *τ*_2 _and *τ*_3 _with *τ*_4_, were modeled to have epistatic effects on CAC values, whereas *τ*_5 _was a major gene with an over-dominant allele for high CAC. In addition to these five genes, three covariates (age, simulated cholesterol values, and simulated high-density lipoprotein (HDL) values) also were modeled to affect CAC values. We considered the M1-M5 SNPs because these SNPs 1) were not involved in the simulation of CAC or any of the CAC related traits (e.g., HDL and cholesterol (CHOL)), 2) were in Hardy-Weinberg equilibrium, and 3) had minor allele frequencies greater than 0.1.

## Methods

### Definition of the CAC phenotype

We first regressed the nonzero CAC values obtained at age less than 60 on age (AGE), CHOL levels, and HDL levels using all of the 6,476 subjects in each of the 200 simulations. The regression coefficients obtained for each given simulation (*b*_*VAR*_; *VAR *= *CHOL*, *HDL*, and *AGE*) were then used to obtain adjusted CAC values at each of the three time points for each individual. The procedures for adjusting CAC values at time *t *were conducted by replicate as follows:

Here, the notation *VAR*_*t *_(for *t *= 1, 2, 3) refers to the value for VAR = *CAC*, *HDL*, *CHOL*, and *AGE *recorded at visit *t*. Then we averaged the three CAC adjusted values (CA60_*t*_) to get the average adjusted CAC values (*CA60*), which were the values we used in the linkage analyses through out this paper.

### Family-based sampling designs

In each case we only sampled unrelated nuclear families for which there was marker data available on both the parents and the siblings of the probands and for which there was simulated CAC data for every sibling. The families denoted as "Random" were randomly chosen. The disease-ascertained families (MI) were obtained by first selecting the disease affected probands, i.e., individuals simulated as having MI events and then including all their siblings. The quantitative-trait-ascertained families (CAC) were obtained similarly to the disease-ascertained families except the probands were ascertained for having adjusted CAC (*CA60*) values of no less than 3,240 (the approximate 90^th ^percentile for these adjusted CAC values for all simulations). Parental genotypes are also obtained for the allele sharing identity-by-decent (IBD) calculation.

In each of the 200 replicates, there were on average about 71.0 unrelated nuclear families (SD = 5.8) with at least one individual having an MI event, and on average about unrelated 90.6 nuclear families (SD = 26.8) with at least one individual having CAC value over 3240. We randomly selected 90 unrelated nuclear families from each simulation so as to have approximately the same number of sibpairs as in the selected samples. We considered each nuclear family with *s *offspring as providing *s*-1 "*effective*" sibpairs whose allele sharing and phenotype differences have a low pairwise correlation for the linkage analysis described below. In order to have a sample of at least 600 effective sibpairs for our analyses, we combined the data from four consecutive simulations into one sample. We then have on average 678 (SD = 26.7) sibpairs from an average of 284 families (SD = 9.7) in each of the 50 combined MI samples, on average 698 (SD = 85.0) sibpairs from an average of 362 families (SD = 46.4) in each combined CAC sample, and 688 sibpairs in 360 families in each combined Random sample.

### Haseman-Elston linkageLinkage analysis

The Haseman-Elston regression-based method [5] was implemented in SAS, which we refer to in this paper as the "univariate" analysis. This method considers the regression equation *Y *= *α *+ *β*_*g*_*I*_*g*_. Here, *Y *denotes the squared difference in CA60 in each sibpair and *I*_*g *_denotes the number of alleles shared IBD at the SNP *g *(for *g *= *τ*_1_, *τ *_2_, *τ *_3_, *τ *_4_, *τ *_5_, *M1*, *M2*, *M3*, *M4*, and *M5*). The Merlin software was used to extract the IBD values of all of the sibpairs at these ten SNPs.

In order to reduce the correlation between squared differences in *CA60 *in sibpairs from the same families, we selected (*s*-1) sibpairs in each family with *s *offspring by pairing the proband with each remaining offspring. In the randomly chosen nuclear families the probands are defined as the individuals having the smallest subject ID numbers among the offspring in the families.

We then did multiple regression extension of the Haseman-Elston method, which we refer to in this paper as the multivariable analysis. Specifically, we considered the regression equation *Y *= *α *+ *β*_*i*_*I*_*i *_+*β*_*j *_*I*_*j *_+ *β*_*ij*_*I*_*i *_*I*_*j*_. Here, *Y *denotes the squared difference in *CA60 *and *I*_*i *_and *I*_*j *_denote the IBD at the SNP *i *and SNP *j *respectively (for {*i*, *j*} = {*τ*_1_, *τ *_2_}, {*τ*_3_, *τ *_4_}, and the 10 pairs from {*M1*, *M2*, *M3*, *M4*, and *M5*}). This extension has been proposed in the past to detect several quantitative trait loci and epistasis [6,7].

Under the null hypothesis of no linkage, the regression coefficients equal 0, i.e., *β*_*i *_= 0, and under the alternative hypothesis, *β*_*i *_< 0. The usual regression *t *statistic (estimated coefficient divided by its estimated standard error) was used to test this hypothesis. These *t *values were converted to LOD scores by setting LOD =  if *t*-value < 0 and LOD = 0 if *t*-value ≥ 0.

### Evaluation of power

The power of the sampling approaches was evaluated by comparing the average  observed in regression-based analyses of each of the CAC-determining SNPs separately and in combination. These were compared with each other and also to the prediction interval estimate for the average  under the null distribution. The empirical null distribution was obtained by using the corresponding regression-based approaches on the same samples to analyze the data on five SNPs (M1-M5) believed to be unrelated to CAC. We decided to focus on the mean and standard deviation of the  under the null hypothesis (rather than obtain empirical critical values for the LOD) because we had only 50 samples per locus per sampling method being considered.

The mean  obtained in univariate linkage analyses of M1-M5 varied from one SNP to the next and from one sampling method to the next, with significant sampling-method-by-SNP interaction. Hence, we pooled the values across five loci for each sampling design to estimate the mean  and then used the results of the variance-components analyses to estimate the variance of the estimated mean under the null hypothesis and the variance of an average observed  obtained using 50 samples. Using this method, the estimated mean  = 0.27 and the 95% prediction interval for the average  obtained based on 50 Random samples at one SNP in the null case would be from 0.00 to 0.76. Similarly, we estimated the mean  = 0.35 and 95% prediction interval from 0.03 to 0.66 for the CAC samples; the mean  = 0.06 and 95% prediction interval from 0.01 to 0.10 for the MI samples.

We obtained a null distribution of the  for the test of interaction by redoing these analyses on the ten pairwise interaction terms generated by considering M1-M5. In this case, the means and estimated prediction interval of the average  based on 50 samples for the test of interaction were as follows: 1) Random samples: mean = 0.30, 95% prediction interval from 0.00 to 0.75; 2) CAC samples: mean = 0.50, 95% prediction interval from 0.00 to 1.14; 3) MI samples: mean = 0.40, 95% prediction interval from 0.00 to 1.06.

We also can derive the asymptotic mean value of  = 0.19 with asymptotic SD = 0.14 in the null case if the assumptions of the regression analysis hold (i.e., independent observations and no collinearity between variables, normally distributed residuals). We do this by noting that the quantity  *  is distributed asymptotically as a 50:50 mixture of 0 and a half-normal random variable (i.e., |*Z*|, where *Z *has standard normal distribution). The significance level associated with a  of 1.73 (or a LOD score of 3.0) is 0.0001. Based on this asymptotic null distribution, our prediction interval for the average  of 50 samples is from 0.15 to 0.23.

### Comparison of sampling designs

We applied two-way ANOVA to determine whether sampling methods were a significant factor and whether the effects of sampling method are the same across loci. Upon observing a significant interaction, the averages of the mean square-root-transformed LODs were compared at each locus using the Scheffe test.

## Results

In Table 1 we reported the mean of the  obtained in the univariate linkage analyses and the mean of the  obtained for the two sets of epistatic loci in the multivariable analyses. We also report the grouping of these means obtained using the Scheffe test. If two means have the same grouping letters then they are not significantly different at the 0.05 level.

**Table 1:** The Scheffe grouping and the mean of  for the five SNPs that determine CAC

SNP	Genetic variance^a^	Scheffe grouping	Mean (+/-)^b^	Sampling design
*τ*_1_		A	0.87+	CAC
		B	0.43+	MI
	0	C	0.05	Random
*τ*2		A	0.64	CAC
		A	0.71+	MI
	1,250	B	0.01	Random
*τ*3		B	0.35	CAC
		A	0.76+	MI
	0	C	0.03	Random
*τ*4		B	0.58	CAC
		B	0.60+	MI
	0	A	1.09+	Random
*τ*5		B	0.48	CAC
		C	0.24+	MI
	10,000	A	1.33+	Random
*τ*_1 _*τ*_2 _epistasis		B	0.13	CAC
		A	0.28	MI
	20,000	B	0.09	Random
*τ*_3 _*τ*_4 _epistasis		A	1.82+	CAC
		A	1.85+	MI
	40,000	B	0.39	Random

^a^Using the mean genetic effects and the population allele frequencies given in Kraja et al [4]. ^b^(+/-): Above (+), below (-), or on () the 95% empirical prediction interval for the average .

We also included in this table the values of the genetic variance attributable to each locus and each interaction. These were calculated from the information on the population allele frequencies and the mean effects of the genetic components given in the Genetic Analysis Workshop 16 Problem 3 answer [4]. According to Haseman and Elston [5], the coefficients of the regression on squared difference on alleles shared IBD are proportional to these genetic variances in samples of unrelated randomly chosen sibpairs. Based on these values, we would expect to observe: 1) lower average  for *τ*_1_, *τ*_3_, and *τ*_4_, 2) higher average  for *τ*_2_, *τ*_5_, and the *τ*_1 _*τ*_2 _interaction, and 3) the highest average  for the *τ*_3 _*τ*_4 _interaction. Because the genetic variance is determined by the joint distribution of the genotypes as well as the effects of the genotypes, these values apply only to the Random samples.

We have denoted those findings which under the alternative are above (+) or below (-) our calculated 95% prediction intervals for SNPs unrelated to CAC for the given sampling method. The values without any sign are on the 95% prediction interval under the null hypothesis. We noted that, with the exception of the *τ*_1_*τ*_2 _epistasis, all of our observed average  are significantly greater than expected using at least one of the sampling methods for each of these CAC-determining SNPs and the *τ*_3_*τ*_4 _epistasis.

We did not show here the results of another multivariable analysis that included all the main effects of *τ*_1_, *τ*_2_, *τ*_3_, *τ*_4_, and *τ*_5 _and the *τ*_1_*τ*_2_, *τ*_3_*τ*_4 _interactions. The findings upon inclusion of all terms were closer to what we would expect in light of the genetic variance attributable to the individual genotypes. That is, the highest observed mean  was observed for the *τ*_3_*τ*_4 _interaction (maximum mean  > 1.8 with both CAC and MI sampling), and *τ*_5 _(maximum mean  = 1.1 using randomly selected sibpairs), with much lower mean  observed for *τ*_1_*τ*_2 _interaction (maximum mean  = 0.25 with MI sampling), *τ*_1 _(maximum mean  = 0.3 with CAC sampling), *τ*_2 _(maximum mean  = 0.3 with CAC sampling), *τ*_3 _(all less than 0.003), and *τ*_4 _(all less than 0.1).

## Discussion and conclusion

We chose to focus on regression-based linkage analyses because they would be computationally feasible for a genome-wide analysis. Furthermore, the extensions to multiple regression allows for us to effectively consider situations where there is epistasis. Interestingly, the multiple-regression linkage analysis approach showed fairly good power to detect the complete epistasis between locus *τ*_3 _and locus *τ*_4 _on the adjusted CAC. These linkage methodologies all are based on the detection of a relationship between phenotype differences in sibpairs and genotype differences in sibpairs. Thus a sample of only sibpairs with large phenotype differences, i.e., discordant sibpairs, would not be appropriate for these analyses.

We expected that both the selection based on quantitative trait (CAC) and the selection based on disease (MI) would show an increase in power over random sampling. However, no one sampling method was consistently the best or the worst. The purpose of selected sampling is to increase the proportion of families with at least one parent heterozygous for the disease-predisposing allele. The allele frequencies of the disease-predisposing allele are 0.5 for all of these loci except the *τ*_5 _locus, where it is 0.2, and hence random sampling results in a substantial proportion (0.4-0.75) of families with at least one heterozygous parent. This may account for our observation in detecting epistasis that the selected samples (MI and CAC) resulted in greater power than the random samples.

A very surprising result was our observation of significantly higher than expected average  using asymptotic theory for the five CAC-unrelated SNPs and the significant between-SNP variance in  observed for these five CAC-unrelated SNPs. Upon taking this variance between SNPs into account, we observe that several values observed under the alternative were not outside of the 95% prediction interval for the null case. We conjecture that the mean  varies considerably in the null case depending on how the families are sampled, the distribution of the number of siblings per family in the sampled families, and the distribution of the quantitative trait.

It was reassuring to observe that the analyses based on MI sibpairs had the same or greater power as the analyses based on the CAC sibpairs. If abnormal levels of the quantitative trait in the absence of the disease were not harmful to one's health, it might be difficult to ascertain individuals with abnormal values. However, if the disease is associated with the high values of this trait, then in identifying a sample of individuals with the disease, we would automatically have a large proportion of individuals with the abnormal values.

We were quite surprised that the univariate linkage analyses of *τ*_5_, the SNP with the highest variance between genotypes (10,000), was not more powerful than that for the other SNPs, which had much lower variance between genotype. On the other hand, when we did a multivariable regression of squared differences on IBD alleles at each locus and included the interaction terms for the two pairs of epistatic SNPs, the magnitudes of the average  for the individual SNPs are in the expected order, with the largest average being observed for *τ*_5_, and lower values for values for the *τ*_1_, *τ*_3_, and *τ*_4_. These findings may indicate that there may be some association between the number of alleles IBD at *τ*_5 _with the number of alleles IBD at these other CAC-determining SNPs or the need to follow up significant single-SNP linkage findings with multivariable analysis.

## List of abbreviations used

AASP: Asymmetrically ascertained sibling pairs; CAC: Coronary artery calcification; CHOL: Cholesterol; HDL: High-density lipoprotein; IBD: Identity-by-decent; MI: Myocardial infarction; SNP: Single-nucleotide polymorphism.

## Competing interests

The authors declare that they have no competing interests.

## Authors' contributions

CH developed the methodology for the study, performed the genetic and statistical analyses, and drafted the manuscript. KL, RSF, S-WC, SHC, and TS performed the data reduction and helped to perform the genetic and statistical analyses. KL and SYS managed the databases and developed a file with the relevant parameters for the analysis. NRM and SJF conceived of the study, participated in its design and coordination, and helped to draft the manuscript. All authors read and approved the final manuscript.
